# Recombineering using RecET in *Corynebacterium glutamicum* ATCC14067 via a self-excisable cassette

**DOI:** 10.1038/s41598-017-08352-9

**Published:** 2017-08-11

**Authors:** Yuanyuan Huang, Lu Li, Shan Xie, Nannan Zhao, Shuangyan Han, Ying Lin, Suiping Zheng

**Affiliations:** 1 Guangdong Key Laboratory of Fermentation and Enzyme Engineering, School of Biology and Biological Engineering, South China University of Technology, Guangzhou, 510006 P. R. China; 2 Guangdong research center of Industrial enzyme and Green manufacturing technology, School of Biology and Biological Engineering, South China University of Technology, Guangzhou, 510006 P. R. China

## Abstract

Gene manipulation is essential for metabolic engineering and synthetic biology, but the current general gene manipulation methods are not applicable to the non-model strain *Corynebacterium glutamicum* (*C*. *glutamicum*) ATCC14067, which is used for amino acid production. Here, we report an effective and sequential deletion method for *C*. *glutamicum* ATCC14067 using the exonuclease-recombinase pair RecE + RecT (RecET) for recombineering via a designed self-excisable linear double-strand DNA (dsDNA) cassette, which contains the Cre*/loxP* system, to accomplish markerless deletion. To the best of our knowledge, this is the first effective and simple strategy for recombination with markerless deletion in *C*. *glutamicum* ATCC14067. This strategy provides a simple markerless deletion strategy for *C*. *glutamicum* and builds a solid basis for producer construction.

## Introduction

*Corynebacterium glutamicum* (*C. glutamicum*) is widely used for the production of L-amino acids, vitamins, organic acids, fuel ethanol and other products^1–3^, and manipulation of its genes is essential for metabolic engineering and synthetic biology^4, 5^. Traditional strategies of counter-selectable systems^6–8^ and Cre*/loxP* site-specific recombination^9–11^ have been successfully used in the model strain of *C. glutamicum* ATCC13032, for gene deletion; however, the genetic manipulation tools are still limited for the non-model strain, such as *C. glutamicum* ATCC14067, which is also widely used to study of amino acid production^12–15^.

The widely used gene deletion approach of the conventional sacB counter-selectable system in *C. glutamicum* ATCC13032 is based on two rounds of homologous recombination, during which only 2% of the events correspond to double-crossover events and which usually requires more than 10 days^16^. Moreover, it does not work in *C. glutamicum* ATCC14067^6, 17^, which may be due to specific or unclear genetic information among different *Corynebacteria* ^12^. The Cre/mutant *loxP* system is useful for sequential gene deletion and large-scale genome engineering in *C. glutamicum* ^9–11^. Cre recombinase can catalyze reciprocal site-specific recombination between the mutated *loxP* sites, *lox71* and *lox66*, and can generate *lox72*, which cannot be recognized by Cre. However, the deletion based on the Cre/mutant *loxP* system involves two plasmids and two rounds of transformation in *C. glutamicum* ^17^, which is also tedious and laborious. Gene deletion via a linear double-strand DNA (dsDNA) cassette with a native recombineering system, which exists in several species^18, 19^, can greatly reduce the workload; however, double-crossover events rarely occur without the integrative vector in *C. glutamicum* ^20^.

The homologous recombination via the phage recombinase has revolutionized bacterial genetics since 1998^21^. It is easy and efficient to construct gene deletion mutants by recombineering via dsDNA in gram-positive and gram-negative bacteria, including *Lactobacillus plantarum* ^22^ and *Pseudomonas syringae* ^23^. The *λ red* system and RecET system from *E. coli* are typical for recombineering^24, 25^. The λ *red* recombination system is composed of three phage-encoded proteins, Exo, Beta and Gam^25^ and the RecET system is composed of only two phage-encoded proteins, RecE and RecT^26^. The proteins λ Exo and RecE can act as the 5′-3′dsDNA-dependent exonuclease that can resect a linear dsDNA to expose a 3′-ended single-stranded DNA (ssDNA) tail; ssDNA-annealing proteins, λ Beta and RecT, which are members of the single-stranded DNA annealing protein (SSAP) family, also called recombinase, bind to the ssDNA tail and promote the annealing of complementary DNA strands, strand exchange and strand invasion.

The RecFACS system (RecT-mediated single-strand recombination via fluorescence-activated cell sorting) is a fast method to introduce genomic mutations and achieves ultrahigh-throughout detection and isolation of productive recombinants in *C. glutamicum* ATCC13032^27^. However, the Lrp-based biosensors can only detect intracellular several amino acids and there are no currently available optical sensors for the most biotechnologically relevant compounds^28^. Therefore, RecFACS is limited for other small moleculars in general and the screening of recombinants is still problematic in *C. glutamicum*. The recombineering with dsDNA needs to be employed in *C. glutamicum* to accomplish effective recombineering and simple selection.

Thus, in this study, we explored the recombination activity of the orthologous exonuclease-recombinase pairs, RecE + RecT (RecET) from Rac phage and Exo + Beta from Lamda phage, and RecET-like pairs, Orf47 + Orf48 from the A118 phage of *Listeria monocytogenes* (*L. monocytogenes*) and OrfB + OrfC from *Legionella pneumophila* (*L. pneumophila*)^29^, in *C. glutamicum* ATCC14067. We also designed a self-excisable linear dsDNA cassette combining the Cre/*loxP* system to perform markerless deletion via RecET recombineering system. This strategy provides a new simple and efficient markerless deletion strategy for *C. glutamicum* ATCC14067.

## Results

### The linear dsDNA recombination efficiency of the different orthologous exonuclease-recombinase pairs in *C. glutamicum* ATCC14067

The orthologous exonuclease-recombinase pairs Exo/Bet, RecE/RecT (RecET), Orf47/Orf48 and OrfB/OrfC, which have been identified to perform the function of linear dsDNA recombineering in *E.coli* ^29^, were selected to test the recombineering activity in *C. glutamicum* ATCC14067. A 0.5 μg linear dsDNA cassette of CrtB/400-Kan was used for the verification (Supplementary Fig. S1). The exonuclease-recombinase pairs of RecET and OrfB/OrfC could catalyze linear dsDNA recombination (Table 1). Orf47/Orf48 showed weak activity, with only ~13 kanamycin resistant colonies, and Exo/Bet did not produce any recombinants in *C. glutamicum* ATCC14067 (Table 1). No recombinant was observed in the control, which contained the linear dsDNA cassette in *C. glutamicum* ATCC14067 without an exonuclease-recombinase pair (Table 1), indicating that linear dsDNA could not lead to recombination in *C. glutamicum* ATCC14067 via the cell’s own recombination system and that the recombination was mediated by the exonuclease-recombinase pairs. The replicative plasmid pZ9, which contained kanamycin resistance cassette, was used to explore the DNA uptake capability, and all transformations produced 3.1 × 10^3^ ~ 3.8 × 10^3^ kanamycin resistant colonies (Table 1), which suggested that expression of the exonuclease-recombinase does not affect DNA uptake. This result is similar to what has been observed for *C. glutamicum* ATCC13032^27^.

**Table 1:** Comparison of the recombination efficiencies of different orthologous exonuclease-recombinase pairs for linear dsDNA.

Vector	Km^r^ per 10^9^ viable cells		
	+dsDNA	−dsDNA	+plasmid
pEC-XC99E	0 ± 0	0	3.1 + 0.21 × 10^3^
pEC-bet/exo	0 ± 0	0	3.8 ± 0.14 × 10^3^
pEC-orf47/orf48	13 ± 2	0	3.6 ± 0.14 × 10^3^
pEC-orfB/orfC	59 ± 3	0	3.7 ± 0.14 × 10^3^
pEC-recE/recT	66 ± 5	0	3.8 ± 0.14 × 10^3^

0.5 μg CrtB/400-Kan cassette or plasmid was used for recombination assay, with a kanamycin concentration of 25 mg L^−1^. All assays were repeated three or more times. Km^r^, kanamycin resistance.

### Optimization of the RecET recombineering

**Figure 1:**
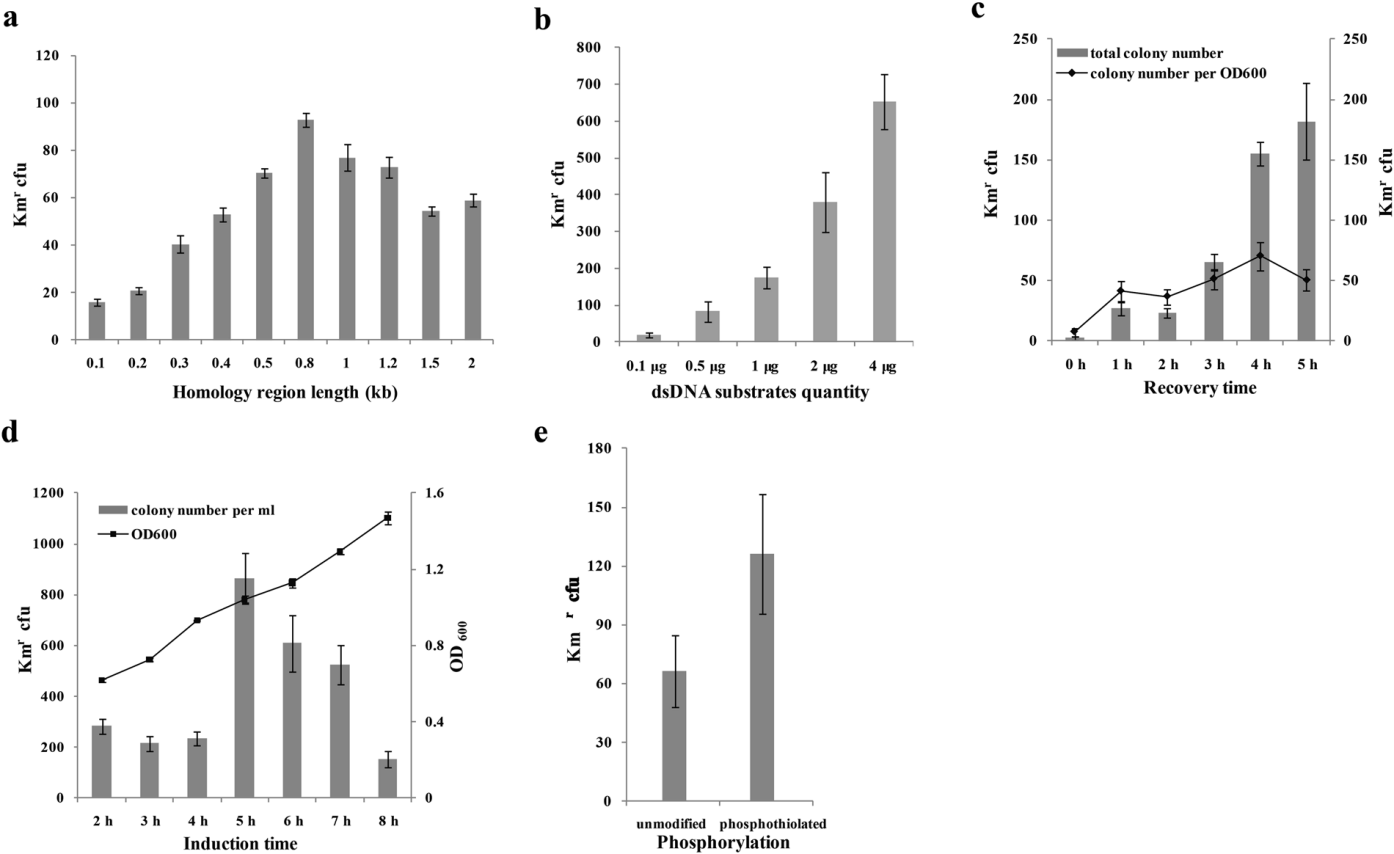
Optimization of linear dsDNA recombineering parameters. (**a**) Effect of the homology length of linear dsDNA on recombination efficiency. 100, 200, 300, 400, 500, 800, 1000, 1200, 1500, 2000 bp left and right homology arms of the *crtB* gene combining with the Kan cassette were used for the investigation. 0.5 μg dsDNA cassettes were used for electroporation. The recovery time was 5 h. (**b**) Effect of the quantity of linear dsDNA on recombination efficiency. 0.1–4 µg CrtB/800-Kan cassettes were used for electroporation. The recovery time was 4 h. (**c**) Effect of the recovery time on recombination efficiency. The recovery time was 0 h to 5 h. 0.5 μg CrtB/800-Kan cassettes were used for electroporation. The total number of colony of per OD_600_ also is shown. (**d**) Effect of the induction time on recombination efficiency. The RecET recombinases were induced for 2–8 h. 0.5 μg CrtB/800-Kan cassettes were used for electroporation and the recovery time was 4 h. OD_600_ of the harvested culture is also shown. (**e**) Effect of the phosphothiolated linear dsDNA on recombination efficiency. 0.5 μg CrtB/800-Kan cassettes were used for electroporation. The recovery time was 4 h. All of the Km^r^ cfu is the number of kanamycin resistance colony per mL. Datas are the means of at least three experiments with standard deviations by error bars.

The RecET pair showed the highest recombination efficiency in *C. glutamicum* ATCC14067, we next varied the conditions to enhance the recombination efficiency. The recombineering frequency of the RecET system is dependent on the length of DNA homology^30^ and the length of homology arms were optimized to improve the linear dsDNA recombination efficiency in *C. glutamicum* ATCC14067. 0.5 µg CrtB-Kan cassettes with the homology lengths from 100 to 2,000 bp (Supplementary Table S1) were used to investigate the change in recombination frequency. The number of kanamycin resistant colonies increased as homology arms length increased from 100 bp to 800 bp (Fig. 1a). Beyond a homology region length of 800 bp, there was no significant increase or even decrease in the number of colony (Fig. 1a), which is similar with the RecTE_Psy_ recombineering in *Pseudomonas syringae* ^31^. The homology arms length may have reached a threshold length for efficient recombineering via RecET.

Then, we assayed the effect of linear dsDNA concentration on recombineering efficiency. 0.1–4 µg CrtB/800-Kan cassettes were used for the electroporation. There was a significant increase in the number of colonies with increasing of dsDNA substrates concentration, with as many as ~653 recombinants when adding the 4 µg CrtB/800-Kan cassettes (Fig. 1b). This result was different from the recombineeing in *Pseudomonas syringae* ^31^ and *Lactobacillus plantarum* ^22^ that the frequency decreased with higher dsDNA substrate. Adding 0.5 µg and 1.0 µg CrtB/800-Kan cassettes achieved ~100 and 200 recombinants and it was suitable for next analysis. So, 0.5 µg or 1.0 µg dsDNA cassettes were used further.

The recovery cultivation time also affected the recombineering efficiency^22^, therefore we varied recovery cultivation time from 0 h to 5 h. The results showed that the total number of recombinants was increased with the increasing recovery time, while the number of colonies per OD_600_ did not obviously increase after 1 h (Fig. 1c). In order to save time and obtain suitable colonies, 4 h recovery time was applicable. To improve the recombineering efficiency, the induction time was optimized (from 2 h to 8 h). The optimal induction time was 5 h, at which point the OD_600_ was ~1.0 (Fig. 1d).

To protect the linear dsDNA from being degraded by a host nuclease, the 5′ end phosphorylated dsDNA could further improve the recombineering efficiency^22, 32^. The dsDNA substrate of CrtB/800-Kan cassette for recombineering was phosphorylated which further improved the recombineering efficiency at least as twice as much in *C.glutamicum* ATCC14067 (Fig. 1e). The results showed that the colony can reach to 1.41 ± 0.18 × 10^3^ colonies per mL (Supplementary Fig. S2) under the condition of 800 bp homology arms, 1 μg phosphorylated dsDNA, 5 h induction time and 4 h recovery time. These work established the RecET recombineering condition in *C. glutamicum* ATCC14067.

### Scheme for markerless deletion of a gene in *C. glutamicum* ATCC14067

The RecET system with linear dsDNA can effectively perform targeted gene replacement by selection marker in *C. glutamicum* ATCC14067. However, a simple selection marker rescue strategy is necessary for metabolic engineering and synthetic biology in *C. glutamicum*. Therefore, we redesigned a self-excisable linear dsDNA cassette containing the site-specific Cre/*loxP* recombination system for recombineering via RecET (RecET-Cre/*loxP* system).

**Figure 2:**
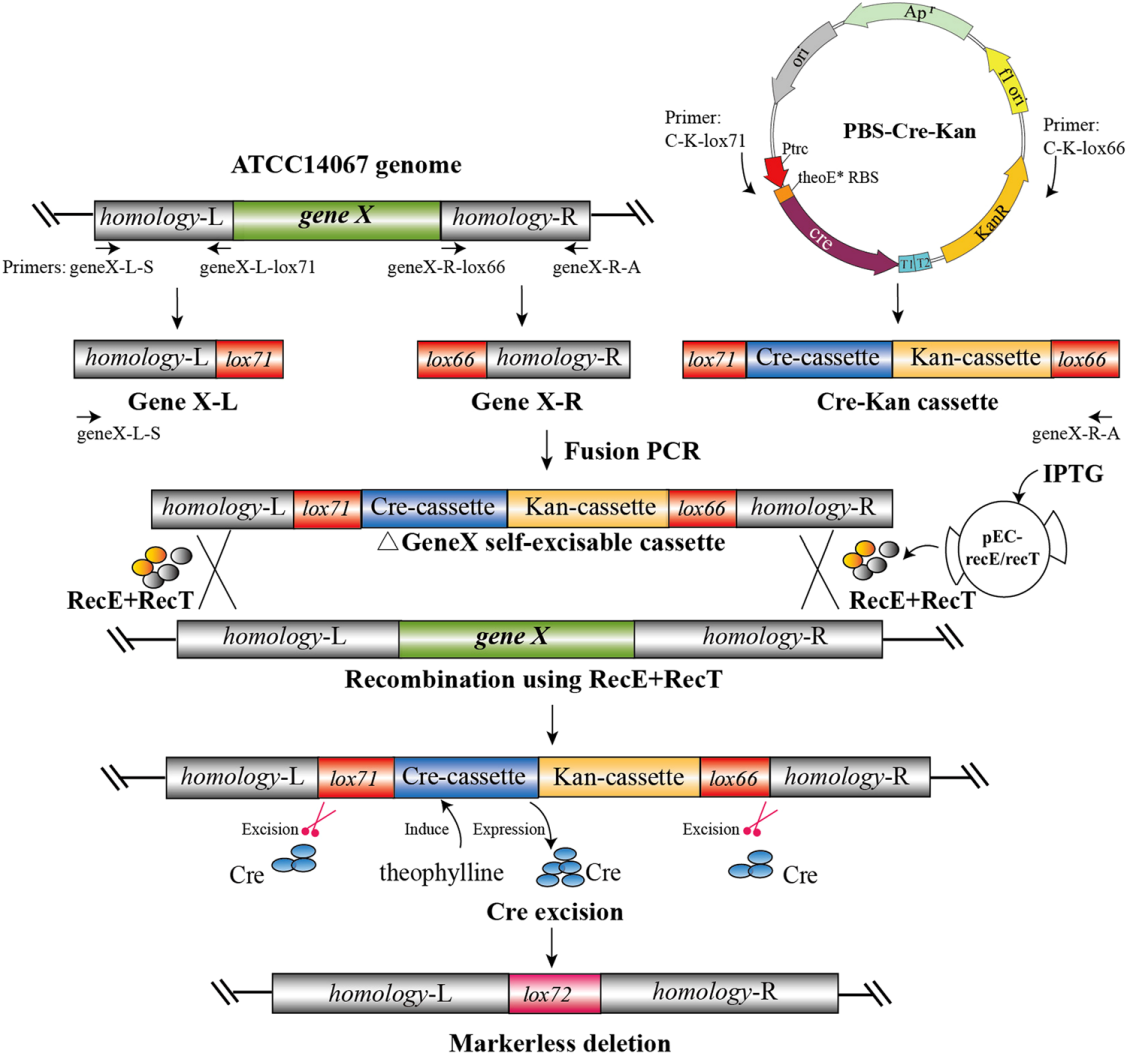
Scheme for gene markerless deletion via a self-excisable cassette in *C. glutamicum* ATCC14067. The theoE*-RBS is theophylline riboswitch E* which can induce the expression of Cre with 1 mM theophylline. The homology-L/R represents the left/right homology arms of the targeted gene, which are approximately 800 bp. GeneX-L represents the targeted gene’s left homology arm and 34 bp *lox71* sequence, and GeneX-R represents the 34 bp *lox66* sequence and the targeted gene’s right homology arm. Cre-Kan cassette contains the 34 bp *lox71* sequence, the Cre expression cassette, the kanamycin resistance cassette and the 34 bp *lox66* sequence. ΔGeneX represents the targeted gene to be deleted. *GeneX* represents the targeted gene. All primers are listed in Supplementary Table S3.

**Figure 3:**
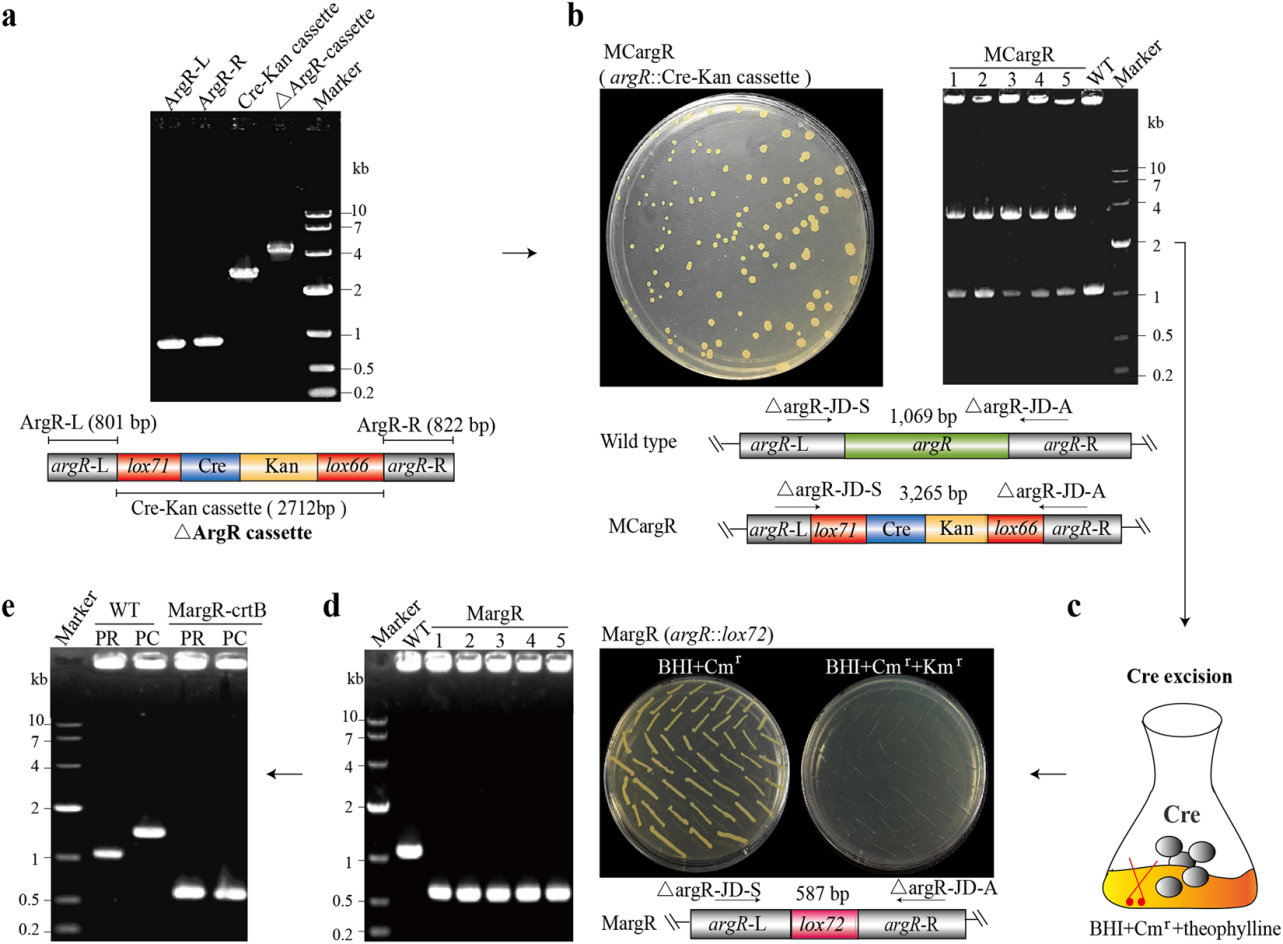
Markerless deletion in *C. glutamicum* ATCC14067. (**a**) ΔArgR-cassette construction. The ΔArgR-cassette contains ArgR-L (*argR* homology left and the 34 bp *lox71* sequence), Cre-Kan cassette and ArgR-R (34 bp *lox66* sequence and *argR* right homology). (**b**) ΔArgR-cassette recombineering with RecET. The primer pairs of ΔargR-JD-S/A were used for the verification of colony PCR. WT is *C. glutamicum* ATCC14067. (**c**) Cre excision by the addition of 1 mM theophylline: at least four recombinants were used to evaluate the excision efficiency. (**d**) Identification markerless deletion of the *argR* gene. MargR represents the strain in which the *argR* gene is replaced by the 34 bp sequence of *lox72*. (**e**) Identification markerless deletion of the *crtB* gene in MargR. MargR-crtB represents the strain in which the *argR* and *crtB* genes are replaced by the sequence of 34 bp *lox72*. PR is the ΔargR-JD-S/A primer pair. PC is the ΔcrtB-JD-S/A primer pair. All primers are listed in Supplementary Table S3. Km^r^, kanamycin resistance. Cm^r^, chloramphenicol resistance.

First, a PBS-Cre-Kan plasmid was constructed containing the Cre expression cassette and kanamycin resistance expression cassette (Fig. 2). The Cre expression cassette is under control of theophylline riboswitch E* (thoE* RBS), which can be induced with 1 mM theophylline (Fig. 2). The kanamycin resistance expression cassette was used as a screening marker. Then, the PBS-Cre-Kan plasmid was used to amplify the generic fragment of Cre-Kan cassette, which had the 34 bp sequence of *lox71* added on the left and *lox66* added on the right, with primers C-K-lox66 and C-K-lox71. The *C. glutamicum* ATCC14067 genome was used for the amplification of ~800 bp left (GeneX-L containing the *lox71* sequence) and right (GeneX-R containing the *lox66* sequence) homologous fragments flanking the gene to be deleted (Fig. 2). Finally, all the three fragments, the generic fragment of Cre-Kan cassette and the left and right homologous fragments, were used for subsequent fusion PCR to generate ~4350 bp linear self-excisable dsDNA cassettes. We named the dsDNA cassettes were ΔArgR*-*cassette, ΔCrtB*-*cassette, ΔNcgl1221*-*cassette and ΔProB*-*cassette, respectively (Fig. 3a and Supplementary Fig. S3). 1 µg of each self-excisable linear dsDNA cassette was transformed into *C. glutamicum* ATCC14067 expressing RecET proteins. The linear self-excisable dsDNA cassettes accomplished the recombination via RecET system in *C. glutamicum* ATCC14067 (Fig. 2). To excise the selection marker and Cre expression cassette, 1 mM theophylline was added to the recombinants to induce the expression of Cre to perform excision between *lox71* and *lox66*, which produced the markerless deletion strain for the subsequent gene deletion (Fig. 2).

### Markerless deletion with the RecET-Cre/*loxP* system

1 µg each of the ΔArgR*-*cassette, ΔCrtB*-*cassette, ΔNcgl1221*-*cassette and ΔProB*-*cassette were transformed into ATCC14067-recE/T competent cells, yielding numbers of Kan-resistant colonies with different gene recombineering (Fig. 3b). At least 30 of the kanamycin resistant colonies were analyzed by PCR using the primer pairs of ΔargR-JD-S/A, ΔcrtB-JD-S/A, ΔNcgl1221-JD-S/A and ΔproB-JD-S/A, respectively (Fig. 3b). At least 94% of the transformants successfully replaced the targeted gene with the Cre-Kan cassette, and none of the correct transformants were mutated by the sequencing (Table 2). The correct recombinants were called MCargR, MCcrtB, MCNcgl1221 and MCproB. These results are in agreement with the linear dsDNA substrate recombineering in *M. smegmatis* and *M. tuberculosis*, where >90% of the drug-resistant colonies correctly replaced the targeted gene^33^. Then, 1 mM theophylline was added to perform marker excision (Fig. 3c). The streak results show that after excision by Cre, the single colonies of the four genes can only grow on plates without kanamycin (Fig. 3d, Supplementary Fig. S4). The PCR results showed that only a ~500 bp fragment was amplified from the single colonies (Fig. 3d, Supplementary Fig. S4). Both of these results indicate that the Cre executed the excision with an efficiency approaching 98% (Table 2), yielding the markerless deletion recombinants MargR, McrtB, MNcgl1 221 and MproB.

**Table 2:** RecET recombineering and Cre excision of different genes in *C. glutamicum* ATCC14067.

Gene targeted	Gene replacement frequency	Mutated frequency	Excision efficiency of Cre
*argR*	94% ± 11	0%	100% ± 0
*crtB*	97% ± 5.8	0%	100% ± 0
*Ncgl1221*	100% ± 0	0%	98% ± 2.0
*proB*	94% ± 11	0%	100% ± 0
*argR- crtB*	97% ± 5.8	0%	100% ± 0
*argR- Ncgl1221*	97% ± 5.8	0%	100% ± 0
*argR- proB*	97% ± 5.8	20%	97% ± 3.8

1 μg targeted linear dsDNA cassettes were used for electroporation and each targeted gene with three experiments performed in parallel. The recovered colonies were analyzed by PCR using at least 10 colonies of each, in parallel, to identify the targeted gene replacement frequency. At least five recombinants were sequenced to identify the mutated frequency. Three or more correct recombinants were induced to explore the Cre excision efficiency.

This method achieved markerless deletion of a single gene, and the MargR (ΔargR::*lox72*) strain was used for the second gene deletion and marker rescue. 1 µg each of the ΔCrtB*-*cassette, ΔNcgl1221*-*cassette and ΔProB*-*cassette were recombined in MargR to generate the new recombinant strains MargR-MCcrtB, MargR-MCNcgl1221 and MargR-MCproB. The PCR results showed that ≥97% of the transformants were correct (Table 2), and the sequencing results showed that none of the transformants were mutated, except for one transformation site of MargR-MCproB (Table 2). The screening and PCR results showed that the second genes of *crtB*, *Ncgl1221*, and *proB* underwent markerless deletion after Cre expression was induced. The double-deletion markerless recombinants MargR-crtB, MargR-MNcgl1221 and MargR-MproB were obtained (Fig. 3e and Supplementary Fig. S5) and Cre excision efficiency exceeded 95% (Table 2). These results are similar to the single gene markerless deletion.

## Discussion

Recombineering with exonuclease-recombinase pairs via linear dsDNA is a powerful method for gene modification. In this study, we employed the RecET recombineering system for dsDNA recombination in *C. glutamicum* ATCC14067. Besides, we developed a system containing a linear self-excisable dsDNA cassette and RecET system (RecET-Cre/*loxP* system). It is the first effective and simple strategy for gene markerless deletion in *C. glutamicum* ATCC14067.

Although recombination activity of RecET was lower than that in *E. coli* ^34^, the recombineering activity can reach up to 1.41 ± 0.18 × 10^3^ colonies per mL in C. *glutamicum* ATCC14067 under optimized recombination conditions (Supplementary Fig. S2). There was no recombinant without the help of RecET, which indicated that the linear dsDNA could’t complete recombination with its own recombination system in *C. glutamicum* ATCC14067, and the RecET from the *E. coli* phage effectively improved the recombination efficiency. It also suggests that the RecET from the *E. coli* phage can across a species barrier to be functional^29^ in *C. glutamicum* ATCC14067.

We observed that the recombineering efficiency of the longer 4.4 kb self-excisible dsDNA of ΔCrtB*-*cassette was significantly lower than the 2.8 kb CrtB/800-Kan dsDNA cassette (Supplementary Fig. S6). Both of them contained the same 800 bp homology arms, but the insertion lengths of the region between the homologous flanking of the two cassettes were 2755 and 1235 bp, respectively (*crtB* gene is 918 bp). This result is consistent with the observation in the RecTE_Psy_ recombineering in *Pseudomonas syringae* ^31^ and the insertion length between the homologous flanking sequences also strongly affects the RecET recombineering efficiency in *C. glutamicum* ATCC14067.

With the RecET-Cre/*loxP* system, the linear dsDNA cassette for recombineering can be generated via two-steps PCR and only requires one round of recombination to obtain the mutated recombinants, which it only takes 2–3 days and the gene replacement frequency can reach up to ≥94%. The subsequent markerless deletion only requires an extra 2–3 days. It is more effective and simpler for recombination in *C. glutamicum* ATCC14067 than the traditional counter-selectable system^16^. This recombineering system also provides a new strategy to perform gene manipulation for *Corynebacteria* and builds a solid basis for producer construction.

## Materials and Methods

### Strains and growth condition

*C. glutamicum* ATCC14067 was grown in BHI liquid medium (37 g L^−1^ brain heart infusion (Becton, Dickinson and company)) at 30 °C, 250 rpm. *E. coli* DH5α was used as the cloning host for plasmid manipulation, and it was cultured in Luria-Bertani medium (10 g L^−1^ peptone, 5 g L^−1^ yeast extract, 10 g L^−1^ NaCl) at 37 °C. If necessary, antibiotics were added in the following concentrations: kanamycin 50 mg L^−1^ (50 Km) or chloramphenicol 15 mg L^−1^ (15 Cm) for *E. coli* and Kanamycin 25 mg L^−1^ (25 Km) and 7.5 chloramphenicol mg L^−1^(7.5 Cm) for *C. glutamicum* ATCC14067. All bacterial strains used in this study are listed in Supplementary Table S2.

### Plasmids and linear dsDNA cassette

The genes of the exonuclease-recombinase pairs are assembled into the *E. coli*-*C. glutamicum* shuttle-inducible vector pEC-XC99E, which can be induced by 1 mM isopropyl-β-d-thiogalactoside (IPTG), to construct the expression plasmids pEC-exo/bet, pEC-orf47/orf48, pEC-orfB/orfC and pEC-recE/recT. PBS-crtB-L/R-Kan plasmid was constructed for the amplification of different homology lengths of linear CrtB-Kan cassettes (Supplementary Fig. S1). The 5′ end phosphorylation of dsDNA was performed by T4 polynucleotide kinase (Thermo Scientific, Waltham, MA).

The theophylline riboswitch E*(theoE*-RBS)^35, 36^ sequence was amplified using the primer pair, theoE-S and theoE-A, from the synthetic theoE*-RBS sequence. Then the *cre* gene and the theoE*-RBS sequence are assembled into pEC-XC99E to construct the plasmid pEC-theoE-cre. For construction of PBS-Cre-Kan, firstly, the DNA fragment of Cre cassette was amplified from plasmid pEC-theoE-cre with primers theoE-Cre-S and theoE-Cre-A. Secondly, the plasmid pK18mobSacB was used for the amplification of the Kan cassette with primers kan-S and kan-A. Finally, the Cre cassette and the Kan cassette are assembled into pBluescript II SK(+) to construct the generic plasmid PBS-Cre-Kan (Supplementary Fig. S7). NEB Builder HiFi DNA Assembly Master Mix (New England BioLabs, Boston, MA) is used to assembly all the plasmids, and the PCR products are generated by KOD DNA polymerase (TOYOBO, Japan). Successful PCR products were confirmed by agarose gel electrophoresis. Plasmid construction and DNA fragments were confirmed by DNA sequencing (Sangon Biotech, Shanghai, China). All plasmids used in this study are listed in Supplementary Table S2. All the primers used in this study are listed in Supplementary Table S3.

### Preparation of the competent cells

A fresh single colony of wild-type *C. glutamicum* ATCC14067, or *C. glutamicum* ATCC14067 carrying pEC-XC99E derivatives encoding recombinases, was inoculated from a BHI plate into fresh BHI liquid medium containing the relevant antibiotic and grown overnight at 30 °C. Each culture was then inoculated into Epo medium (5 g L^−1^ yeast, 10 g L^−1^ tryptone, 10 g L^−1^ NaCl, 4 g L^−1^ isoniazide, 25 g L^−1^ glycine, 1 g L^−1^ Tween80) containing the relevant antibiotic with or without 1 mM IPTG at an initial OD_600_ of 0.3. When the cultures reached an OD_600_ of ~0.9, the cells were collected to make competent cells. The cells were cooled on ice for 15 min and then harvested at 4,000 rpm at 4 °C for 10 min. Following washed twice in 30 ml of ice-cold 10% (v/v) glycerol, cells were suspended in 1/70 (v/v) 10% glycerol.

### Recombineering assay

80 μl of fresh unfrozen electrocompetent cells carrying the exonuclease-recombinase pairs or pEC-XC99E mixed with linear dsDNA cassette and incubated on ice for 5 min. Then the cell-plasmid DNA/dsDNA mixture was transferred to an ice-cold electroporation cuvette (0.1-cm electrode gap). Electroporation was performed with a Bio-Rad Micropulser set by three times 1.8 KV/cm (Ec1) pulse. The cell-plasmid DNA/dsDNA mixture was inoculated into 6 ml of the resuscitation medium LBHIS (2.5 g L^−1^ yeast, 5 g L^−1^ tryptone, 5 g L^−1^ NaCl, 18.5 g L^−1^ Brain Heart Infusion, 91 g L^−1^ sorbitol) containing the relevant antibiotics. Then cells were placed in a hot bath for 6 min at 46 °C and incubated for hours at 30 °C, 250 rpm. LBHIS-Km25-Cm7.5 solid medium was used for recombinant selection and the cells were cultured at 30 °C for 36~48 h for colony-forming unit (cfu) determination. For exonuclease-recombinase pairs’ recombination activity analysis, the empty plasmid pEC-XC99E with linear dsDNA was used as a positive control. The exonuclease-recombinase pairs’ expression plasmids without the addition of linear dsDNA were used as a negative control. The replicative plasmid pZ9, which contains kanamycin resistant, was used to explore the competence for uptake efficiency of DNA.

### Cre excision assay

The Cre expression cassette is under the control of thoE*-RBS, which can be induced by the addition of 1mM theophylline. To allow Cre-mediated intermolecular excision and accomplish the deletion of kanamycin resistance selection marker, the correct transformants were inoculated into BHI medium containing 1 mM theophylline and cultured for 24 h, at 30 °C, 250 rpm. The cells were then streaked onto the BHI-Cm7.5 plate with 1 mM theophylline and incubated at 30 °C for 16 h. Then, the single colonies were streaked into the BHI-Cm7.5-Km25 and BHI-Cm7.5 plate to explore the excision efficiency. To further identification the makerless excision, PCR identification and sequencing were used for the single colonies which can only grow on plates without kanamycin (BHI-Cm7.5).

## Electronic supplementary material

Supplementary information
