# Supplementary material for: Recombineering using RecET in Corynebacterium glutamicum ATCC14067 via a self-excisable cassette
Source: Sci Rep. 2017 Aug 11;7:7916. doi: 10.1038/s41598-017-08352-9 (PMC5554157; doi:10.1038/s41598-017-08352-9)
Supplement: Supplementary file 1 — Supplementary information [file 41598_2017_8352_MOESM1_ESM.pdf]

Recombineering using RecET in *Corynebacterium glutamicum* ATCC14067

via a self-excisable cassette

SUPPLEMENTARY INFORMATION

Yuanyuan Huang[1, 2], Lu Li[1, 2], Shan Xie[1, 2], Nannan Zhao[1, 2], Shuangyan Han[1, 2], Ying Lin[1, 2], Suiping Zheng[1, 2]*

(1)Guangdong Key Laboratory of Fermentation and Enzyme Engineering, School of Biology and Biological Engineering, South China University of Technology, Guangzhou, 510006, P. R. China

(2)Guangdong research center of Industrial enzyme and Green manufacturing technology, School of Biology and Biological Engineering, South China University of Technology, Guangzhou, 510006, P. R. China

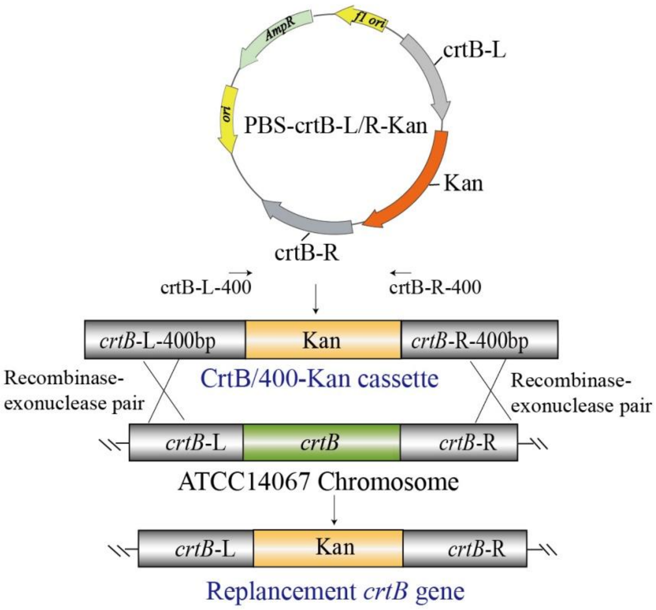

Fig S1. Construction of linear dsDNA cassettes for recombineering optimization. 400 bp is the length of homology arms for recombineering, and the length of 100, 200, 300, 400, 500, 800, 1000, 1200, 1500 and 2000 bp homology arms of cassettes for *crtB* gene replacement are constructed with the same method in this study. Kan is the kanamycin resistance cassette. All primers are listed in Supplementary Table S3.

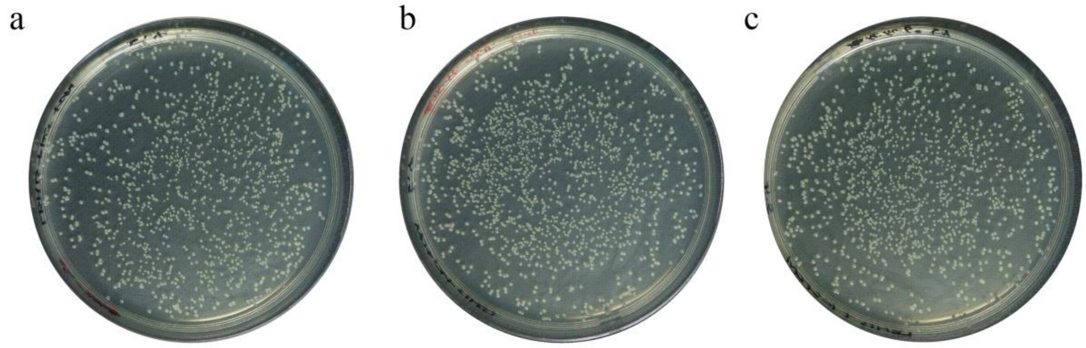

Fig S2. RecET Recombineering at the optimized conditions. 1.0 μg phosphothiolated CrtB/800-Kan cassette was used for recombineering. The homology length is ~800 bp. The induce time is 5 h with an initial OD_{600} of 0.3. The recovery time is 4 h and 1 mL cultures were spread onto the BHIS-Kan25-Chl7.5 solid medium.

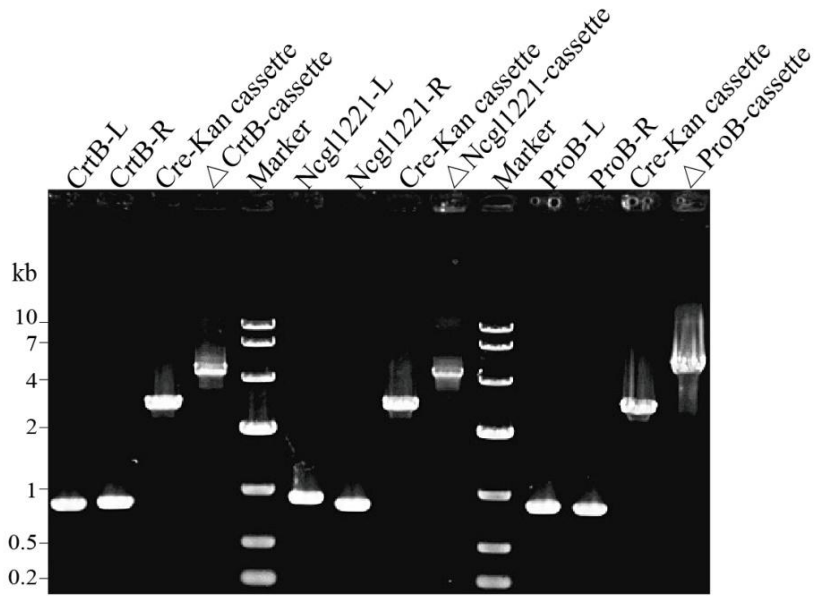

Fig S3. Construction of the self-excisable linear dsDNA cassettes. The ΔCrtB-cassette contains CrtB-L (800 bp *crtB* left homology and the 34 bp *lox71* sequence), the Cre-Kan cassette and CrtB-R (the 34 bp *lox66* sequence and 810 bp *crtB* right homology). The ΔNcgl1221-cassette contains Ncgl1221-L (845 bp *Ncgl1221* left homology and 34 bp *lox71* sequence), the Cre-Kan cassette and Ncgl1221-R (34 bp *lox66* sequence and 786 bp *Ncgl122*1 right homology). The ΔProB-cassette contains ProB-L (817 bp *proB* left homology and 34 bp *lox71* sequence), the Cre-Kan cassette and ProB-R (34 bp *lox66* sequence and 809 bp *proB* right homology).

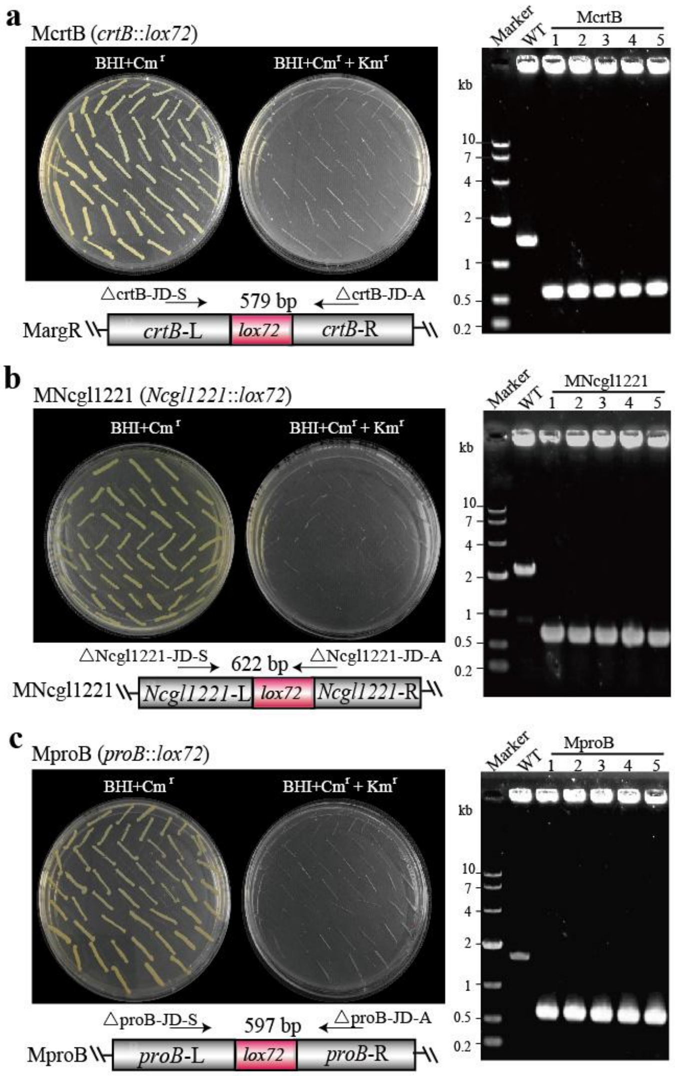

Fig S4. Identification of the markerless single gene deletion. (a) McrtB represents the strain in which the *crtB* gene is replaced by the 34 bp *lox72* sequence. (b) MNcgl1221 represents the strain in which the *Ncgl1221* gene is replaced by the 34 bp *lox72* sequence. (c) MproB represents the strain in which the *proB* gene is replaced by the 34 bp *lox72* sequence. Km^r is the kanamycin resistance and Cm^r is the chloramphenicol resistance. All primers are listed in Supplementary Table S3.

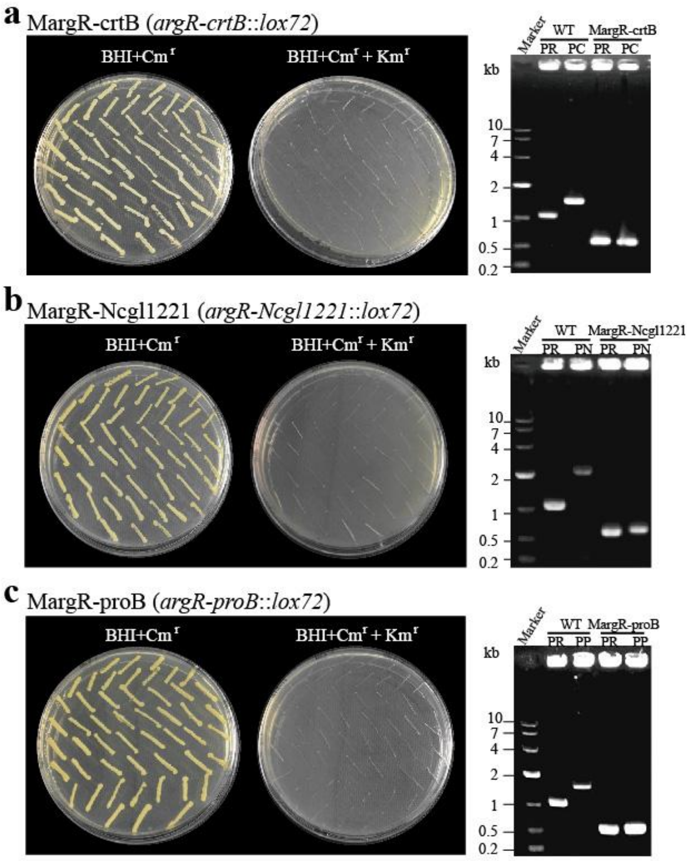

Fig S5. Identification of the markerless double gene deletion. (a) MargR-crtB represents the strain in which the genes *argR* and *crtB* are replaced by the 34 bp *lox72* sequence. PR is the argR-JD-S/A primer pair. PC is the crtB-JD-S/A primer pair. (b) MargR-Ncgl1221 represents the strain in which the genes *argR* and *Ncgl1221* are replaced by the 34 bp *lox72* sequence. PR is the argR-JD-S/A primer pair. PN is the Ncgl1221-JD-S/A primer pair. (c) MargR-proB represents the strain in which the genes *argR* and *proB* are replaced by the 34 bp *lox72* sequence. PR is the argR-JD-S/A primer pair. PP is the proB-JD-S/A primer pair. All primers are listed in Supplementary Table S3. Km^r is the kanamycin resistance and Cm^r is the chloramphenicol resistance.

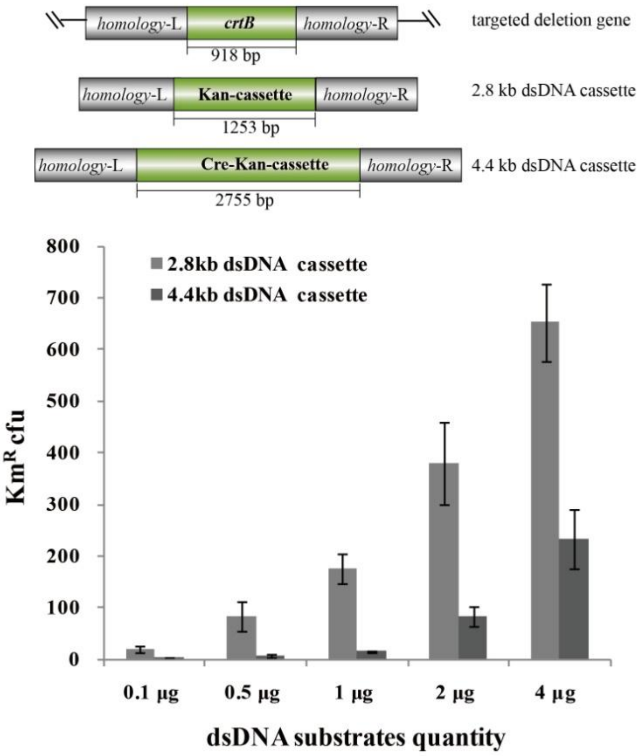

Fig S6. Effect of the quantity of linear dsDNA and the insertion length on recombination efficiency. 0.1-4 μg linear dsDNA of 2.8-kb CrtB/800-Kan cassette and 4.4-kb ΔCrtB-cassette were used for recombineeing, respectively. The *crtB* gene targeted for deletion is 918 bp. 2.8-kb CrtB/800-Kan and 4.4-kb CrtB-cassette contain the same left/right homology arm and the length of the region between the homologous flanking of the two cassettes are 1253 and 2755 bp, respectively.

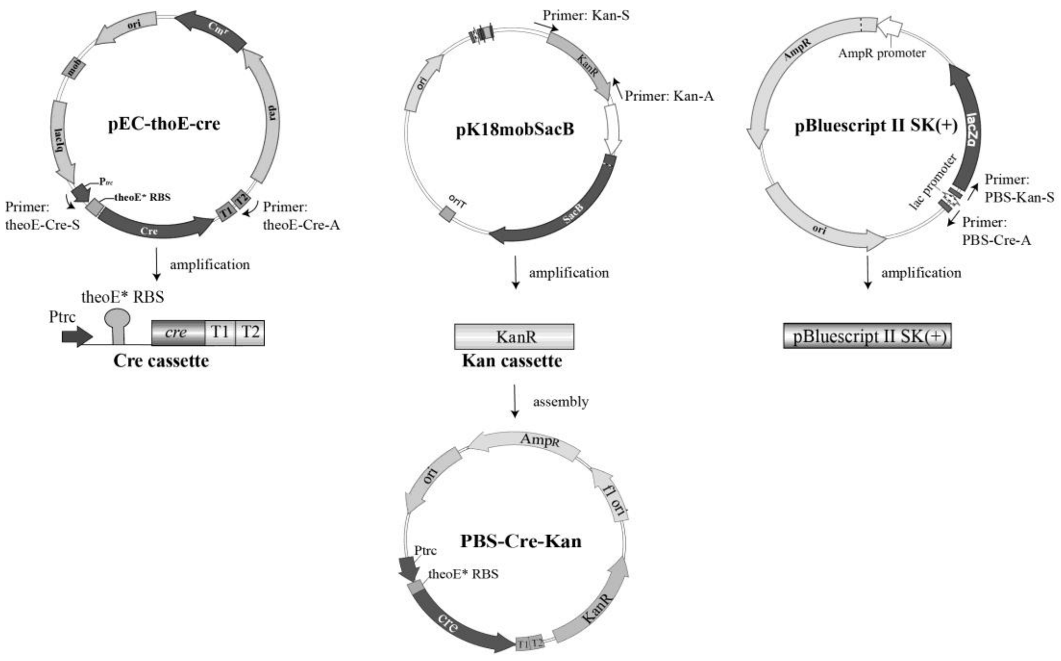

Fig S7. Construction of the generic plasmid PBS-Cre-Kan. The Cre expression cassette contains the trc promoter sequence, theoE*-RBS sequence which control the expression of Cre and can be induced with 1mM theophylline, cre gene coding sequence and terminator of T1 and T2 sequence.

Table S1. Linear self-excisable dsDNA cassettes used in this study

dsDNA cassettes	Relevant characteristics
CrtB/400-Kan cassette	The cassette contains 400 bp left and right homology arms of *crtB* gene and Kan cassette (kanamycin resistance cassette)
CrtB/100-Kan cassette	The cassette contains 100 bp left and right homology arms of *crtB* gene and Kan cassette (kanamycin resistance cassette)
CrtB/200-Kan cassette	The cassette contains 200 bp left and right homology arms of *crtB* gene and Kan cassette (kanamycin resistance cassette)
CrtB/300-Kan cassette	The cassette contains 300 bp left and right homology arms of *crtB* gene and Kan cassette (kanamycin resistance cassette)
CrtB/500-Kan cassette	The cassette contains 500 bp left and right homology arms of *crtB* gene and Kan cassette (kanamycin resistance cassette)
CrtB/800-Kan cassette	The cassette contains 800 bp left and right homology arms of *crtB* gene and Kan cassette (kanamycin resistance cassette)
CrtB/1000-Kan cassette	The cassette contains 1000 bp left and right homology arms of *crtB* gene and Kan cassette (kanamycin resistance cassette)
CrtB/1200-Kan cassette	The cassette contains 1200 bp left and right homology arms of *crtB* gene and Kan cassette (kanamycin resistance cassette)
CrtB/1500-Kan cassette	The cassette contains 1500 bp left and right homology arms of *crtB* gene and Kan cassette (kanamycin resistance cassette)
CrtB/2000-Kan cassette	The cassette contains 2000 bp left and right homology arms of *crtB* gene and Kan cassette (kanamycin resistance cassette)
ΔArgR-cassette	The cassette contains the 801 bp left homology arms of *argR* gene, the Cre-Kan cassette and the 822 bp right homology arms of *argR* gene
ΔCrtB-cassette	The cassette contains the 800 bp left homology arms of *crtB* gene, the Cre-Kan cassette and the 810 bp right homology arms of *crtB* gene
ΔNcgl1221-cassette	The cassette contains the 845 bp left homology arms of *Ncgl1221* gene, the Cre-Kan cassette and the 786 bp right homology arms of *Ncgl1221* gene
ΔProB-cassette	The cassette contains the 792 bp left homology arms of *proB* gene the Cre-Kan cassette and the 809 bp right homology arms of *proB* gene

Table S2. Strains and plasmids used in this study

Strains or plasmids	Relevant characteristics	Sources or references
**Strains**		
E.coli DH5α	F$^-$ φ80*lac*ZΔM15 Δ(*lac*ZYA-*arg*F) U169 *rec*A1 *end*A1 *hsd*R17 (r$_k$-, m$_k$+) *pho*A *sup*E44 λ$^-$ *thi*$^-$1 *gyr*A96 *rel*A1	Buy from Invitrogen
ATCC14067	*Corynebacterium glutamicum* ATCC14067	Buy from ATCC
14067-XC99E	Cm^r, ATCC14067 derivative harboring the expression vector PEC-XC99E	This work
14067- exo/bet	Cm^r, ATCC14067 derivative harboring the expression vector PEC-exo/bet	This work
14067- orf47/48	Cm^r, ATCC14067 derivative harboring the expression vector PEC-orf47/orf48	This work
14067- orfB/C	Cm^r, ATCC14067 derivative harboring the expression vector PEC-orfB/orfC	This work
14067-recE/T	Cm^r, ATCC14067 derivative harboring the expression vector PEC-recE/recT	This work
MCargR	Cm^r, Km^r, *argR* gene is replaced by the Cre-Kan cassette sequence in 14067-recE/T	This work
MCcrtB	Cm^r, Km^r, *crtB* gene is replaced by the Cre-Kan cassette sequence in 14067-recE/T	This work
MCNcgl1221	Cm^r, Km^r, *Ncgl1221* gene is replaced by the Cre-Kan cassette sequence in 14067-recE/T	This work
MCproB	Cm^r, Km^r, *proB* gene is replaced by the Cre-Kan cassette sequence in 14067-recE/T	This work
MargR	Cm^r, *argR* gene markerless deletion mutant harboring the expression vector PEC-recE/recT	This work
McrtB	Cm^r, *crtB* gene markerless deletion mutant harboring the expression vector PEC-recE/recT	This work
MNcgl1221	Cm^r, *Ncgl1221* gene markerless deletion mutant harboring the expression vector PEC-recE/recT	This work
MproB	Cm^r, *proB* gene markerless deletion mutant harboring the expression vector PEC-recE/recT	This work
MargR-MCcrtB	Cm^r, Km^r, *crtB* gene is replaced by the Cre-Kan cassette sequence in MargR	This work
MargR-MCNcgl1221	Cm^r, Km^r, *Ncgl1221* gene is replaced by the Cre-Kan cassette sequence in MargR	This work
MargR-MCproB	Cm^r, Km^r, *proB* gene is replaced by the Cre-Kan cassette sequence in MargR	This work
MargR-McrtB	Cm^r, *crtB* gene markerless deletion mutant in MargR	This work
MargR-MNcgl1221	Cm^r, *Ncgl1221* gene markerless deletion mutant in MargR	This work
MargR-MproB	Cm^r, *proB* gene markerless deletion mutant in MargR	This work
**Plasmids**		
pZ9	Km^r, pMLB1034::ϕ (zwf'-'!acZY') (-35 to +118) (Hyb) (PQ-)	[1]
pEC-XC99E	Cm^r, shuttle expression vector of *E. coli*/*C. glutamicum*	[2]
pEC-bet/exo	Cm^r, pEC-XC99E derivative for IPTG induced expression of *bet* and *exo* from phage lamda of *E.coli*	This work
pEC-orf47/orf48	Cm^r, pEC-XC99E derivative for IPTG induced expression of *orf47*and *orf48* from phage of *Listeria monocytogenes*	This work
pEC-orfB/orfC	Cm^r, pEC-XC99E derivative for IPTG induced expression of *orfB* and *orfC*from *Legionella pneumophila*	This work
pEC-recE/recT	Cm^r, pEC-XC99E derivative for IPTG induced expression of *recE* and *recT* from Rac prophage from *E.coli*	This work
pBluescript II SK(+)	Ap^r, Cloning vector	Stratagene
PBS-crtB-L/R-Kan	Ap^r, Km^r, pBluescript II SK(+) derivative for amplification of the CrtB-Kan cassettes with different length of homology arms	This work
pEC-theoE-cre	Cm^r, pEC-XC99E derivative for amplification of the Cre expression cassette (induced by theophylline)	This work
PBS-Cre-Kan	Ap^r, Km^r, pBluescript II SK(+) derivative for amplification the Cre-Kan cassette	This work

Km^r, kanamycin resistance. Cm^r, chloramphenicol resistance. Ap^r, ampicillin resistance.

Table S3. Primers used in this study

Primers	DNA Sequence (5'-3')
bet/exo-S	CAAAAAGGAGGCCCTTCAGATGAGTACTGCACTCGCAACG
bet/exo-A	GGCACCAATAACTGCCTTAATCATCGCCATTGCTCCCCAAA
recT/recE-S	CAAAAAGGAGGCCCTTCAGATGAGCACAAAACCACTCTTC
recT/recE-A	GGCACCAATAACTGCCTTAATTATTCCTCTGAATTATCGA
orfC/orfB-S	CAAAAAGGAGGCCCTTCAGATGACAAAATTATGTTTTAGTG
orfC/orfB-A	GGCACCAATAACTGCCTTAATTAAGCCTTATCCTGATTAGT
orf48/orf47-S	CAAAAAGGAGGCCCTTCAGATGGCTATTGCAAAAGAAAAGAC
orf48/orf47-A	GGCACCAATAACTGCCTTAATTAGATCATTGACCCTTGAACC
PEC-A	CTGAAGGGCCTCCTTTTTGTTATCCG
PEC-S	TTAAGGCAGTTATTGGTGCCCATGCG
crtB-R-A	CATCGCCTATGCTCGAAATACTGC
crtB-R-S	TCATAGCTGAGCCTGCTTCTGGTA
crtB-L-S	TAATAGGTACGCCAGACCAAAAGG
crtB-L-A	ATGAAGACATCAACTACAACTCCA
kan-crtB-A	AGAAGCAGGCTCAGCTATGAATGGGTTAAAAAGGATCGATCCTC
kan-crtB-S	GTTGTAGTTGATGTCTTCATCGCAAGCGCAAAGAGAAAGCAGGT
PBS-crtB-A	TTGGTCTGGCGTACCTATTATTGTTATCCGCTCACAATTCCACAC
PBS-crtB-S	ATTTCGAGCATAGGCGATGATGCAGGAATTCGATATCAAGCTTA
crtB-R-100	CGCCTTCTTGGTGCCTGCTTTGTT
crtB-R-200	CCGCAACGTTCGCAGCACTTTATG
crtB-R-300	CGTATCCGTGAAGAAACCAAAACC
crtB-R-400	TCACGTTTTCAATTTCCCAGGAGCAGACCCCGC
crtB-R-500	TCATGCTGTTGCTGCGGTCTATTGTCGCACCAC
crtB-R-800	CTGACTCCCCCTCTGGCTCA
crtB-R-1000	ACTAAAAGCGGATGGTGTGTCCCA
crtB-R-1200	CCAGCCCTGCTATTTGTATTCGGT
crtB-R-1500	ATCCTGCTGCTTTCGGGTCAAACT
crtB-R-2000	AGGTACAGGAACAGTTGGGAGATTTCAC
crtB-L-100	CATCACGTGCTAAAAGTGCAGAAG
crtB-L-200	GGTCCGGTATCCCATCGAAAGCCA
crtB-L-300	ATAACCAGGCGTTAATTCTACC
crtB-L-400	GTTTTGCACCCGCGCCGGGTTCAATGGATT
crtB-L-500	TCCCGGTGAAGCAGCGGGCCCAACGTGGAGAAATT
crtB-L-800	GAAGTGTTGATAGAAATGACCTCAG
crtB-L-1000	AGAGGATTAATACCGCTCCAATTC
crtB-L-1200	TAAAACAAAAAGGTTTTCGTATCCAGCAGGTGCAACG
crtB-L-1500	GGTAGCACCGGCATAGAAGAGGTT
crtB-L-2000	CATAATGCTGCTGCCGAGGTAA
cre-A	CAATAACTGCCTTAACTAATCGCCATCTTCCAG
cre-S	TAAGGAGGCAACAAGATGTCCAATTTACTGACC
PEC-cre-A	TTCCACACATTATACGAGCCGGATG
PEC-theoE-S	TTAAGGCAGTTATTGGTGCCCATGCGA
theoE-A	CAGTAAATTGGACATCTTGTTGCCTCCTTAGCA
theoE-S	GTATAATGTGTGGAAGAGTACAACTCACGATAG
theoE-template	GAGTACAACTCACGATAGATTCCGGTGATACCAGCATCGTCTTGATGCCCTTGG CAGCACCCTGCTAAGGAGGCAACAAG
PBS-Cre-A	GCGACACGAATTATGCAGTTTGTTATCCGCTCACAATTC
PBS-Kan-S	TCGATCCTTTTTAACCCATTGCAGGAATTCGATATCAAG
Kan-S	AATTAATTCCGCGCAAGCGCAAAGAGAAAGCA
Kan-A	ATGGGTTAAAAAGGATCGATCCTC
theoE-Cre-S	ACTGCATAATTCGTGTCGCTCAAG
theoE-Cre-A	CTTTGCGCTTGCGCGGAATTAATTCATGAGCG
C-K-lox66	TACCGTTCGTATAATGTATGCTATACGAAGTTATATGGGTTAAAAAGGATCGAT CC
C-K-lox71	TACCGTTCGTATAGCATACATTATACGAAGTTATACTGCATAATTCGTGTCGCT CA
argR-L-S	TCAACTCGTACTCGCTTCTCCTTC

argR-L-lox71	TGCAGTATAACTTCGTATAATGTATGCTATACGAACGGTAGTCTTACCTCGGCTGGTTGGCC
argR-R-lox66	ACCCATATAACTTCGTATAGCATACATTATACGAACGGTAAGCGCCCCTAGTTCAAGGCTTG
argR-R-A	TGATGACCTCATCTGGAGCGTTAC
crtB-L-S2	GAAGTGTTGATAGAAATGACCTCA
crtB-L-lox71	TGCAGTATAACTTCGTATAATGTATGCTATACGAACGGTAATGAAGACATCAACTACAACTCC
crtB-R-lox66	ACCCATATAACTTCGTATAGCATACATTATACGAACGGTATCATAGCTGAGCCTGCTTCTGG
crtB-R-A2	GTCACTAGTGCTGACTCCCCCTCT
Ncgl-L-S	GACGGTGGTGACTTTTGAACGAAG
Ncgl-L-lox71	TGCAGTATAACTTCGTATAATGTATGCTATACGAACGGTAGACGCTGATTACAGACGTGTCC
Ncgl-R-lox66	ACCCATATAACTTCGTATAGCATACATTATACGAACGGTAGAGCCAAGATTAGCGCTGAAAAGTAG
Ncgl-R-A	GAACGGAGTCCAAGTTTGCATCAG
proB-L-S	GATGGCCCGTGTTCATTATCTCCG
proB-L-lox71	TGCAGTATAACTTCGTATAATGTATGCTATACGAACGGTATCCGGATTCATGTCCGTATGCG
proB-R-lox66	ACCCATATAACTTCGTATAGCATACATTATACGAACGGTAAGCGCGGGCCTGCTGGTGGCAG
proB-R-A	GTCAGGAAGTGGCTCAGGATC
ΔargR-JD-A	AGGTCGAGAGAAACTGCGATGACTTCAC
ΔargR-JD-S	GTTGTCATCACCGATACCTGGGTATCCA
ΔcrtB-JD-A	GGTCTGACAGTAACCGGCGGAGTAATTA
ΔcrtB-JD-S	CCAGGCGTTAATTCTACCAAATCGAGAT
ΔNcgl-JD-A	TGCGACAGATGTCTGTTGCAAAGTTGGC
ΔNcgl-JD-S	TTGAAGGCGTAGTTTGCGGTGATGCCCT
ΔproB-JD-A	GCGCCAGAATTAAACCAATGGTGGACTC
ΔproB-JD-S	CAGGTCTACGTGTATACGATGGTAACGC

References

1. Fawcett, W. P. & Jr, W. R. Purification of a MalE-SoxS fusion protein and identification of the control sites of *Escherichia coli* superoxide-inducible genes. **14**, 669-679 (1994).
2. Kirchner, O. & Tauch, A. Tools for genetic engineering in the amino acid-producing bacterium *Corynebacterium glutamicum*. *Journal of biotechnology* **104**, 287-299 (2003).
